# Interpretable machine learning of non-traditional lipid indices for diagnostic classification of CHD in patients with comorbid MASLD and T2DM: a multicenter study

**DOI:** 10.3389/fnut.2026.1803996

**Published:** 2026-04-22

**Authors:** Luo Lv, Yuli Guo, Yubo Ren, Bao Li, Xiaofang Li, Qinghua Han, Haixiong Wang

**Affiliations:** 1 Shanxi Medical University, Taiyuan, Shanxi, China; 2 First Clinical Medical College of Shanxi Medical University, Taiyuan, Shanxi, China; 3 Department of Cardiology, The Second Hospital of Shanxi Medical University, School of Medicine, Shanxi Medical University, Taiyuan, China; 4 Department of Digestive Oncology, Shanxi Bethune Hospital, Tongji Shanxi Hospital, Third Hospital of Shanxi Medical University, Taiyuan, Shanxi, China; 5 Department of Cardiology, The First Hospital of Shanxi Medical University, Taiyuan, China; 6 Department of Cardiology, Cardiovascular Hospital Affiliated to Shanxi Medical University, Shanxi Key Laboratory of Heart Failure Precision Medicine, Shanxi Cardiovascular Hospital (Institute), Shanxi Clinical Medical Research Center for Cardiovascular Disease, Taiyuan, Shanxi, China

**Keywords:** Castelli risk index-II, coronary heart disease, machine learning, MASLD, type 2 diabetes mellitus

## Abstract

**Background:**

Patients with comorbid metabolic dysfunction-associated steatotic liver disease (MASLD) and type 2 diabetes mellitus (T2DM) have a significantly heightened risk for coronary heart disease (CHD). Conventional lipid profiles often underestimate residual cardiovascular risk. This study identifies valuable non-traditional lipid indicators and develops an interpretable machine learning framework for CHD identification in this population.

**Methods:**

This multicenter retrospective study analyzed 1,823 patients with MASLD and T2DM. Following 1:1 propensity score matching, 630 participants were used for association analysis, whereas the complete unmatched Cohort I (n = 1,665) was used for machine learning model development, with an independent cohort of 158 patients for external validation. Logistic regression and restricted cubic spline (RCS) models evaluated associations between CHD risk and eight non-traditional lipid indices. Six machine learning algorithms were compared using cascaded feature selection, with model transparency provided by Shapley Additive Explanations (SHAP) and Local Interpretable Model-agnostic Explanations (LIME).

**Results:**

Multivariable analysis revealed that all eight non-traditional lipid indices were significantly associated with CHD risk, with Castelli risk index-II (CRI-II) demonstrating the strongest independent association (OR = 2.394, 95% CI: 2.065–2.788). RCS analysis identified linear positive associations for CRI-II, while non-traditional indices such as remnant cholesterol (RC), atherogenic index of plasma (AIP), and lipoprotein combined index (LCI) exhibited significant nonlinear associations with CHD risk. Furthermore, CRI-II showed the highest positive correlation with the severity of coronary lesions as quantified by the Gensini score (*ρ* = 0.302, *p* < 0.001). The final Stacking ensemble model incorporated 10 variables. This model showed competitive and relatively balanced performance, with AUCs of 0.750 and 0.756 and Brier scores of 0.156 and 0.191 in the internal test set and the independent external validation cohort, respectively. SHAP and LIME analyses further indicated that CRI-II, eGFR, age, and LCI were the major drivers of model classification.

**Conclusion:**

Non-traditional lipid indices, particularly CRI-II, showed strong associations with CHD in patients with concomitant MASLD and T2DM. Integrating these indicators into an interpretable machine learning framework provides a robust and transparent tool for CHD identification, potentially facilitating early clinical evaluation and decision-making.

## 1 Introduction

Metabolic dysfunction-associated steatotic liver disease (MASLD) and Type 2 diabetes mellitus (T2DM) represent significant global public health challenges, frequently co-existing and synergistically increasing the risk of cardiovascular complications (1, 2). This clinical synergy is underpinned by shared pathophysiological mechanisms, a fact reinforced by the 2023 multi-society Delphi consensus which transitioned the nomenclature from non-alcoholic fatty liver disease (NAFLD) to MASLD (3). By requiring the presence of at least one cardiometabolic risk factor, such as T2DM, the new MASLD definition explicitly recognizes that systemic metabolic dysfunction is the primary driver of both hepatic and extrahepatic progression. This metabolic milieu fosters a pro-atherogenic state, which in turn leads to coronary heart disease (CHD) as a primary cause of mortality in this population (4, 5). Despite advancements in diagnostic and therapeutic strategies over recent decades, this population continues to face a disproportionately high risk of cardiovascular events, which markedly reduces quality of life and imposes a substantial economic burden on healthcare systems (6, 7).

Dyslipidemia is a pivotal driver of atherosclerosis and is closely associated with the severity of coronary artery stenosis (8, 9). While traditional lipid parameters, such as low-density lipoprotein cholesterol (LDL-C) and total cholesterol (TC), are widely utilized in clinical practice, they often fail to comprehensively capture the complex atherogenic risk in individuals with MASLD and T2DM, who exhibit high metabolic heterogeneity. In recent years, several non-traditional lipid-derived indices, including the Castelli risk index I and II (CRI-I, CRI-II), atherogenic index of plasma (AIP), atherogenic coefficient (AC), lipoprotein combined index (LCI), remnant cholesterol (RC), non-high-density lipoprotein cholesterol (Non-HDL-C), and the RC/HDL-C ratio, have been proposed as more sensitive biomarkers for cardiovascular risk (10). These indices can be calculated from routine laboratory parameters, potentially facilitating CHD identification and supporting individualized clinical evaluation. However, the specific associations between these non-traditional lipid indices and the risk of CHD, as well as the severity of coronary artery stenosis, remain insufficiently characterized in the population with comorbid MASLD and T2DM.

Accurate identification of CHD in this high-risk group remains a formidable challenge. Most traditional clinical assessment tools rely predominantly on conventional risk factors and linear statistical methods, showing a limited capacity to capture the complex, non-linear interactions between metabolic derangements and lipid-derived biomarkers (11). Furthermore, the lack of integration of non-traditional lipid indices limits the applicability of existing models in data-driven clinical decision-making (12). Machine learning (ML) methods, with their ability to process high-dimensional data and capture complex non-linear associations, have demonstrated superior discriminative performance compared with traditional statistical methods (13–15). To address the “black-box” nature of ML models, explanatory techniques such as Shapley Additive Explanations (SHAP) and Local Interpretable Model-agnostic Explanations (LIME) have been introduced to provide global insights and individual-level decision transparency (16, 17). Nevertheless, many existing ML studies are limited by single-center data, small sample sizes, or a lack of external validation, which hinders their clinical integration and generalizability.

To overcome these limitations, we conducted a multi-center retrospective study using electronic medical records (EMRs) from two tertiary hospitals. We systematically evaluated the associations between eight non-traditional lipid indices and both CHD risk and coronary lesion severity. Building on these findings, we utilized a cascaded feature selection strategy to develop and compare six machine learning algorithms, with robustness evaluated through internal testing and independent external validation. Ultimately, this study aims to identify the most clinically valuable non-traditional lipid predictors and provide a robust, transparent, and practical tool for CHD identification in this high-risk population.

## 2 Materials and methods

### 2.1 Research design and ethical considerations

The study was conducted as a multicenter retrospective cohort investigation utilizing EMR systems from two tertiary medical centers, specifically the Shanxi Cardiovascular Hospital and the Second Hospital of Shanxi Medical University, with screening spanning from January 2023 to December 2023. The investigation adhered strictly to the principles within the Declaration of Helsinki and was conducted and reported in full accordance with the Strengthening the Reporting of Observational Studies in Epidemiology (STROBE) guidelines (18, 19). The research protocol received formal approval from the institutional review boards of both hospitals. Given the nature of the study as a retrospective analysis of de-identified data, the requirement for written informed consent from the participants was waived by the ethics committees.

### 2.2 Study population and eligibility criteria

Inclusion criteria for this study required participants to be at least 18 years of age, have a confirmed diagnosis of MASLD complicated by T2DM, and have undergone coronary angiography (CAG) based on clinical indications. Exclusion criteria included prior use of statins or triglyceride (TG)-lowering medications; a history of CHD, percutaneous coronary intervention (PCI), or coronary artery bypass grafting (CABG); and other major cardiac conditions, including rheumatic heart disease, valvular disease, severe congenital heart disease, cardiomyopathy, cardiac syndrome X, or severe heart failure. In addition, participants with malignancies, autoimmune diseases, acute or chronic infections, severe cerebrovascular accidents, or missing data on the standard lipid profile were excluded. Ultimately, Cohort I (*n* = 1,665) served as the primary study cohort for clinical characteristic association analysis and diagnostic classification model development, while Cohort II (*n* = 158) was utilized as an independent external validation cohort to evaluate model generalizability.

### 2.3 Disease definitions

MASLD was diagnosed according to the 2023 multi-society Delphi consensus, requiring hepatic steatosis on abdominal ultrasound plus at least one cardiometabolic risk factor (3). Because all participants had T2DM, the cardiometabolic risk criterion was automatically satisfied; thus, MASLD was confirmed after excluding excessive alcohol consumption (≥210 g/week for men and ≥140 g/week for women), viral hepatitis, and other known causes of liver injury (3). T2DM was ascertained from electronic medical records by any of the following: a discharge diagnosis of T2DM, prior use of glucose-lowering medications, fasting blood glucose (FBG) ≥ 7.0 mmol/L, or hemoglobin A1c (HbA1c) ≥ 6.5% (20). CHD was defined as at least 50% stenosis of a major coronary artery or its significant branches, as documented in discharge records and confirmed by coronary angiography (21). The Gensini score was subsequently employed to quantify the severity and extent of coronary artery disease based on the degree of narrowing and the anatomical location of stenosis identified during coronary angiography (22).

### 2.4 Data collection and preprocessing

Detailed clinical data collected within 24 h of admission included demographics, anthropometric measurements, clinical risk factors, and vital signs. Laboratory assessments comprised routine blood tests, inflammatory markers, glucose metabolism indices, a standard lipid profile, liver and kidney function tests, cardiac biomarkers, coagulation parameters, and thyroid function. Echocardiographic parameters and Gensini scores were also recorded. In addition, eight non-traditional atherogenic lipid indices, including the AIP, non-HDL-C, AC, CRI-I, CRI-II, LCI, RC, and the RC/HDL-C ratio, were computed for subsequent association analysis and model development. AIP = log10(TG/HDL-C), non-HDL-C = TC—HDL-C, AC = (TC—HDL-C)/HDL-C, CRI-I = TC/HDL-C, CRI-II = LDL-C/HDL-C, LCI = (TC × TG × LDL-C)/HDL-C, RC = TC - HDL-C - LDL-C, RC/HDL-C = RC/HDL-C.

During data preprocessing, variables with more than 30% missingness were excluded. The remaining missing values were handled using multiple imputation by chained equations (MICE) to preserve the inherent correlations among features. For the association analysis only, propensity score matching (PSM) was performed in Cohort I to balance the CHD and non-CHD groups on age, sex, body mass index (BMI), hypertension, and smoking status, using 1:1 nearest-neighbor matching with a caliper of 0.05 and targeting a standardized mean difference (SMD) < 0.10.

### 2.5 Statistical analysis

Continuous variables were summarized as mean ± standard deviation for normally distributed data and as median (interquartile range) for non-normally distributed data. Categorical variables were presented as counts (percentages). Between-group comparisons were conducted using the Student’s t test, Mann–Whitney U test, or chi-square test, as appropriate. To evaluate the associations between lipid indices and the risk of CHD, multivariable logistic regression was performed using three hierarchical models: the unadjusted model included only the exposure variable; Model I adjusted for age, sex, BMI, smoking status, and hypertension; and Model II additionally adjusted for HbA1c, creatinine, white blood cell count, systolic blood pressure, diastolic blood pressure, alanine aminotransferase, and albumin. Restricted cubic spline (RCS) models, implemented via the rcssci package, were further applied to examine potential nonlinear dose–response relationships, with knots placed at the 5th, 35th, 65th, and 95th percentiles. Spearman correlation analysis was used to assess the relationships between lipid indices and the severity of coronary lesions, quantified by the Gensini score. All analyses were conducted using R (version 4.5.0) and Python (version 3.12). A two-sided *p* value < 0.05 was considered statistically significant.

### 2.6 Feature selection and model development

Feature selection and model development were conducted in the complete Cohort I (*n* = 1,665), which was randomly divided into training and test sets in a 7:3 ratio. To avoid potential data leakage and ensure that the model focused on the independent diagnostic value of demographic, metabolic, and lipid-related variables, hs-cTnI and NT-proBNP were excluded from the candidate feature set. A cascaded, multi-step feature selection pipeline was implemented. First, a LightGBM classifier was used for initial screening, and the top 30 variables ranked by feature importance were retained. Subsequently, hierarchical clustering addressed multicollinearity by removing lower-ranked redundant variables from each cluster. Next, sequential forward selection (SFS) with stratified five-fold cross-validation was performed to rank candidate features according to their incremental contribution to model performance, and the top 10 features were ultimately selected for model development. We trained multiple classifiers, including random forest (RF), XGBoost, LightGBM, logistic regression (LR), support vector machine (SVM), and a Stacking ensemble model with RF, XGBoost, and LightGBM as base learners and LR as the meta-learner. Bayesian hyperparameter optimization was performed using Optuna, and stratified five-fold cross-validation was rigorously employed during training to improve robustness and reduce overfitting. For each model, the optimal classification threshold was selected on the training data to achieve a balance between sensitivity and specificity, and the same threshold was subsequently applied to the internal test set and the external validation cohort.

The final model was evaluated in the internal test set and the external validation cohort (Cohort II) using the area under the receiver operating characteristic curve (AUC), accuracy, sensitivity, specificity, positive predictive value (PPV), negative predictive value (NPV), F1 score, Brier score, and decision curve analysis (DCA). To enhance interpretability and mitigate the “black-box” nature of complex models, we applied complementary explainability methods. SHAP was used to quantify global feature contributions and to summarize overall feature–outcome relationships. In parallel, LIME provided case-level, local approximations to clarify the decision logic for individual predictions. In addition, to characterize potentially nonlinear associations between key features and CHD risk, SHAP dependence plots were generated, and locally weighted scatterplot smoothing (LOESS) was applied to fit smoothed trend lines with 95% confidence intervals.

## 3 Results

### 3.1 Cohort construction and baseline characteristics

**Figure 1:**
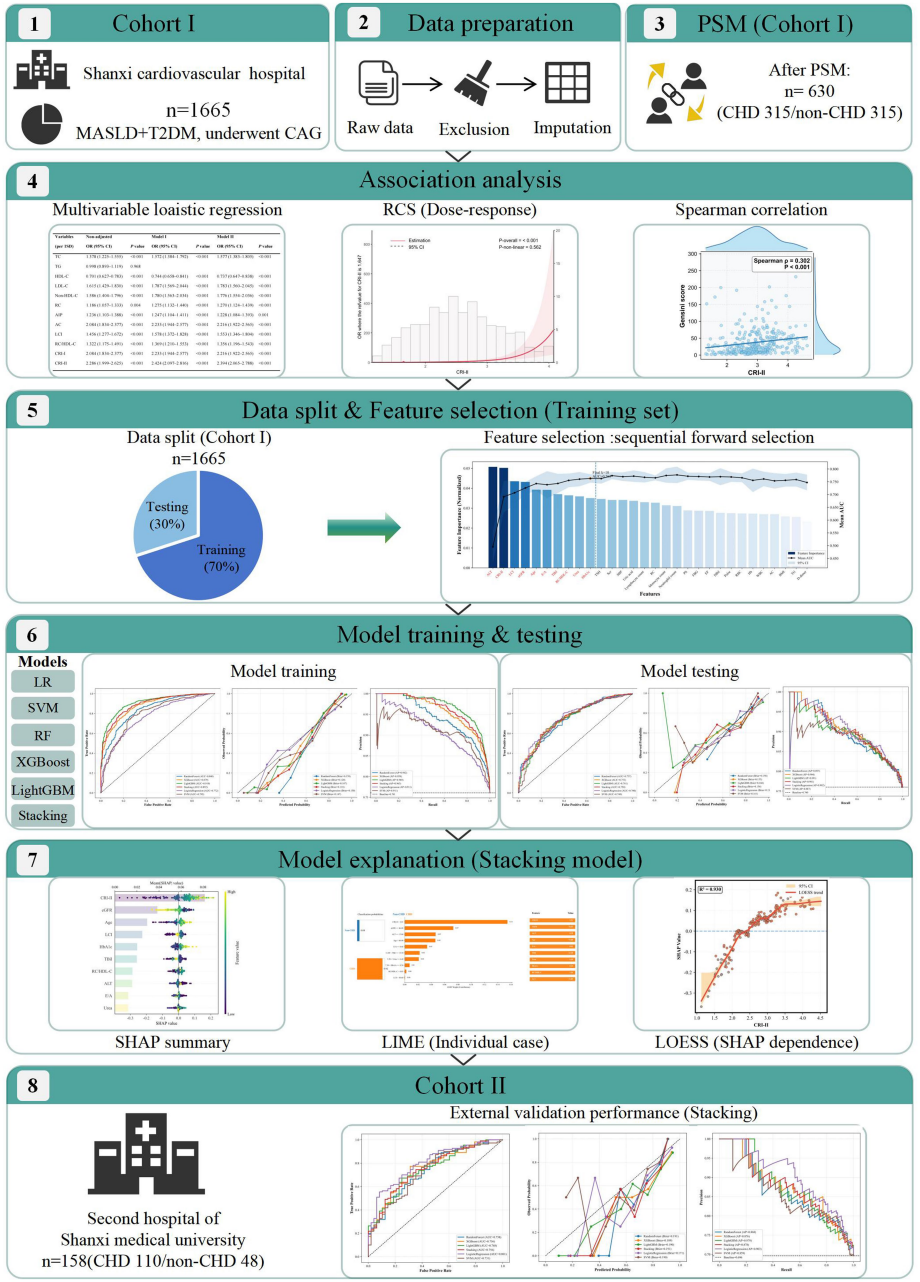
Workflow of non-traditional lipid index identification and interpretable machine learning model development. The workflow illustrates the systematic process of cohort construction, data preprocessing, association analysis, feature selection, model training, and interpretability analysis.

A total of 1,823 patients with MASLD and concomitant T2DM were included, and the study flow is shown in Figure 1. Cohort I initially comprised 1,665 patients. For the association analysis, 1:1 PSM was performed in Cohort I to reduce confounding, yielding a matched cohort of 630 covariate-balanced participants, including 315 patients with CHD and 315 without CHD. Post-matching comparisons showed no significant between-group differences in key baseline characteristics, including age, sex, BMI, hypertension, and smoking history (all *p* > 0.05; Table 1). Supplementary Figure S1 further demonstrated adequate covariate balance, with SMDs <0.10 for all matched variables. Based on this matched cohort, we evaluated the associations between lipid indices and CHD risk. For the machine learning analysis, the complete Cohort I (*n* = 1,665) was randomly divided into a training set and an internal test set at a ratio of 7:3. Cohort II included 158 patients (110 with CHD and 48 without CHD) without matching and served as an independent quasi-external validation cohort. Baseline characteristics of the training set, test set, and validation cohort are provided in Supplementary Table S1.

**Table 1:** Baseline characteristics of the study population stratified by coronary heart disease status before and after propensity score matching.

Characteristics	Before PSM			After PSM		
	CHD (*n* = 1,266)	Non-CHD (*n* = 399)	*P* value	CHD (*n* = 315)	Non-CHD (*n* = 315)	*P* value
Age (years)	61.96 (8.98)	59.13 (8.97)	<0.001	60.52 (8.60)	60.24 (8.53)	0.686
Sex (male)	542 (42.8)	91 (22.8)	<0.001	76 (24.1)	78 (24.8)	0.926
BMI (kg/m^2^)	26.92 (3.21)	26.70 (3.23)	0.238	26.85 (3.60)	26.61 (3.13)	0.381
Smoke (%)	390 (30.8)	60 (15.0)	<0.001	50 (15.9)	53 (16.8)	0.829
HT (%)	914 (72.2)	242 (60.7)	<0.001	199 (63.2)	200 (63.5)	1.000
SBP (mmHg)	135.08 (16.81)	131.43 (15.47)	<0.001	133.75 (16.99)	131.96 (15.41)	0.167
DBP (mmHg)	80.29 (9.82)	80.54 (10.08)	0.662	80.17 (9.94)	80.51 (10.05)	0.670
Pulse (beats/min)	72.84 (11.44)	73.83 (10.54)	0.127	71.46 (10.86)	74.06 (10.62)	0.002
RBC (10^9^/L)	4.62 (0.49)	4.50 (0.48)	<0.001	4.53 (0.47)	4.52 (0.48)	0.736
WBC (10^9^/L)	6.51 (1.61)	6.28 (1.61)	0.015	6.52 (1.60)	6.23 (1.60)	0.021
Plt (10^9^/L)	218.75 (54.49)	223.50 (55.83)	0.131	224.88 (54.01)	221.87 (55.56)	0.490
FBG (mmol/L)	7.06 (2.35)	6.64 (1.86)	0.001	7.29 (2.31)	6.67 (1.88)	<0.001
HbA1C (%)	7.70 (1.40)	7.35 (1.18)	<0.001	7.82 (1.41)	7.36 (1.16)	<0.001
LDL_C (mmol/L)	2.62 (0.68)	2.32 (0.63)	<0.001	2.85 (0.71)	2.31 (0.62)	<0.001
TC (mmol/L)	4.24 (0.91)	3.97 (0.88)	<0.001	4.50 (0.92)	3.95 (0.86)	<0.001
TG (mmol/L)	1.97 (0.85)	1.98 (1.07)	0.968	2.05 (0.88)	1.98 (1.09)	0.410
HDL_C (mmol/L)	0.98 (0.20)	1.06 (0.22)	<0.001	0.98 (0.18)	1.06 (0.22)	<0.001
Alt (U/L)	24.35 (14.01)	25.56 (14.72)	0.139	24.81 (14.20)	25.22 (13.67)	0.712
Alb (g/L)	41.64 (3.33)	41.75 (3.43)	0.568	41.26 (3.19)	41.80 (3.41)	0.040
TB (μmol/L)	19.40 (12.91)	20.07 (14.07)	0.377	18.93 (13.11)	19.36 (13.31)	0.679
Scr (mmol/L)	63.16 (14.10)	59.42 (13.04)	<0.001	59.93 (13.27)	60.01 (12.79)	0.943
Uric acid (μmol/L)	322.00 (86.80)	312.15 (84.90)	0.047	320.77 (81.53)	313.13 (84.18)	0.248
Urea (mmol/L)	5.60 (1.66)	5.32 (1.62)	0.003	5.47 (1.71)	5.38 (1.65)	0.505
NT-proBNP (pg/mL)	59.50 [30.00, 122.00]	46.00 [24.00, 84.00]	<0.001	68.00 [35.00, 150.50]	48.00 [24.00, 85.00]	<0.001
hs-cTnI (ng/L)	3.62 [2.37, 6.06]	2.79 [1.88, 4.00]	<0.001	3.85 [2.34, 8.46]	2.90 [1.90, 4.00]	<0.001
APTT (s)	30.84 (3.38)	30.58 (3.03)	0.163	30.58 (3.11)	30.75 (2.98)	0.484
EF (%)	65.49 (6.67)	65.66 (6.13)	0.652	65.74 (6.57)	65.65 (6.14)	0.851
LA (mm)	36.09 (3.57)	35.50 (3.57)	0.004	35.58 (3.42)	35.45 (3.53)	0.631
E/A ratio	0.81 (0.27)	0.86 (0.30)	0.006	0.83 (0.29)	0.83 (0.28)	0.773

Data are presented as mean ± standard deviation (SD) for normally distributed continuous variables, median [interquartile range, IQR] for non-normally distributed continuous variables, or number (percentage) for categorical variables. *p*-values were calculated using Student’s *t*-test, Mann–Whitney U test, or Chi-square test, as appropriate. PSM, propensity score matching; CHD, coronary heart disease; BMI, body mass index; HT, hypertension; SBP, systolic blood pressure; DBP, diastolic blood pressure; RBC, red blood cell; WBC, white blood cell; Plt, platelets; FBG, fasting blood glucose; HbA1c, glycated hemoglobin; LDL-C, low-density lipoprotein cholesterol; TC, total cholesterol; TG, triglycerides; HDL-C, high-density lipoprotein cholesterol; ALT, alanine aminotransferase; Alb, albumin; TB, total bilirubin; Scr, serum creatinine; NT-proBNP, N-terminal pro-B-type natriuretic peptide; hs-cTnI, high-sensitivity cardiac troponin I; APTT, activated partial thromboplastin time; EF, ejection fraction; LA, left atrial diameter.

Data quality assessment indicated low missingness in Cohort I, with 38 of 49 variables having missing rates below 5%. In the external validation cohort (Cohort II), missingness across the selected features was ≤11.39%, and approximately half of the variables had missing rates below 5%, supporting overall data reliability (Supplementary Table S2). Baseline biochemical comparisons showed that, relative to the non-CHD group, patients with CHD had significantly higher white blood cell counts, FBG, HbA1c, TC, LDL-C, albumin, and cardiac biomarkers (NT-proBNP and hs-cTnI), accompanied by lower pulse rate and HDL-C levels (all *p* < 0.05; Table 1).

### 3.2 Associations of lipid indices with CHD risk and dose–response relationships

**Figure 2:**
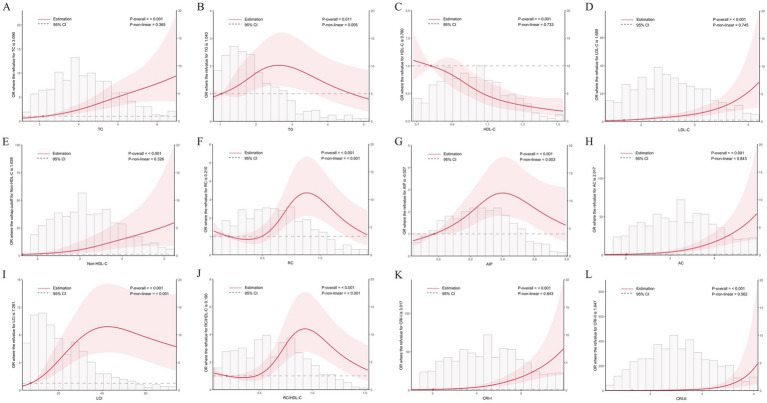
Dose–response relationships between lipid indices and the risk of coronary heart disease based on restricted cubic spline. **(A)** TC, **(B)** TG, **(C)** HDL-C, **(D)** LDL-C, **(E)** Non-HDL-C, **(F)** RC, **(G)** AIP, **(H)** AC, **(I)** LCI, **(J)** RC/HDL-C, **(K)** CRI-I, and **(L)** CRI-II.

Multivariable logistic regression analyses (Table 2) showed that triglycerides (TG) were not significantly associated with CHD risk in the unadjusted model (*p* = 0.968). In contrast, higher TC and LDL-C levels were significantly and positively associated with CHD risk, whereas HDL-C was significantly inversely associated. Notably, all eight non-traditional lipid-derived indices included in the analyses—AIP, non-HDL-C, AC, LCI, RC, the RC/HDL-C ratio, CRI-I, and CRI-II—showed significant positive associations with CHD risk (all *p* < 0.01). These associations remained robust after extensive adjustment (Model II), with CRI-II demonstrating the strongest independent association (OR = 2.394, 95% CI: 2.065–2.788). RCS analyses further characterized the dose–response patterns (Figure 2). TC, LDL-C, non-HDL-C, AC, CRI-I, and CRI-II showed approximately linear positive associations with CHD risk (*P*_overall < 0.001; *P*_non-linear > 0.05), whereas HDL-C exhibited a significant linear inverse association. In contrast, TG, RC, AIP, LCI, and the RC/HDL-C ratio displayed significant nonlinear inverted U-shaped relationships with CHD risk (*P*_non-linear < 0.01).

**Table 2:** Univariate and multivariate logistic regression analyses of the association between lipid indices and the risk of coronary heart disease.

Variables (per 1SD)	Non-adjusted		Model I		Model II	
	OR (95% CI)	*P* value	OR (95% CI)	*P* value	OR (95% CI)	*P* value
TC	1.378 (1.225–1.555)	<0.001	1.572 (1.384–1.792)	<0.001	1.577 (1.383–1.805)	<0.001
TG	0.998 (0.893–1.119)	0.968				
HDL-C	0.701 (0.627–0.783)	<0.001	0.744 (0.658–0.841)	<0.001	0.737 (0.647–0.838)	<0.001
LDL-C	1.615 (1.429–1.830)	<0.001	1.787 (1.569–2.044)	<0.001	1.783 (1.560–2.045)	<0.001
Non-HDL-C	1.586 (1.404–1.796)	<0.001	1.780 (1.563–2.034)	<0.001	1.776 (1.554–2.036)	<0.001
RC	1.186 (1.057–1.333)	0.004	1.275 (1.132–1.440)	<0.001	1.270 (1.124–1.439)	<0.001
AIP	1.236 (1.103–1.388)	<0.001	1.247 (1.104–1.411)	<0.001	1.228 (1.084–1.393)	0.001
AC	2.084 (1.834–2.377)	<0.001	2.233 (1.944–2.577)	<0.001	2.216 (1.922–2.565)	<0.001
LCI	1.456 (1.277–1.672)	<0.001	1.578 (1.372–1.828)	<0.001	1.553 (1.346–1.804)	<0.001
RC/HDL-C	1.322 (1.175–1.491)	<0.001	1.369 (1.210–1.553)	<0.001	1.356 (1.196–1.543)	<0.001
CRI-I	2.084 (1.834–2.377)	<0.001	2.233 (1.944–2.577)	<0.001	2.216 (1.922–2.565)	<0.001
CRI-II	2.286 (1.999–2.625)	<0.001	2.424 (2.097–2.816)	<0.001	2.394 (2.065–2.788)	<0.001

Odds ratios (ORs) and 95% confidence intervals (CIs) represent the risk of CHD per 1-SD increment in each lipid index. Model adjustments: The Non-adjusted model included only the specific lipid index. Model I was adjusted for age, sex, BMI, smoking, and hypertension. Model II was further adjusted for HbA1c, Scr, WBC, SBP, DBP, ALT, and albumin.

In addition, Spearman correlation analyses (Supplementary Figure S2) indicated that CRI-II had the strongest positive correlation with the severity of coronary lesions, as assessed by the Gensini score (*ρ* = 0.302, *p* < 0.001). CRI-I, AC, LCI, and AIP were also positively correlated with the Gensini score (all *p* < 0.05), whereas HDL-C was significantly negatively correlated (ρ = −0.228, *p* < 0.001). Although several associations reached statistical significance, the overall correlation coefficients were weak to moderate in magnitude (ρ ≤ 0.302). By contrast, traditional lipid measures (TC, TG, and LDL-C) and several non-traditional indices (non-HDL-C, RC, and the RC/HDL-C ratio) were not significantly correlated with the Gensini score (all *p* > 0.05).

### 3.3 Feature selection and machine-learning model development

**Figure 3:**
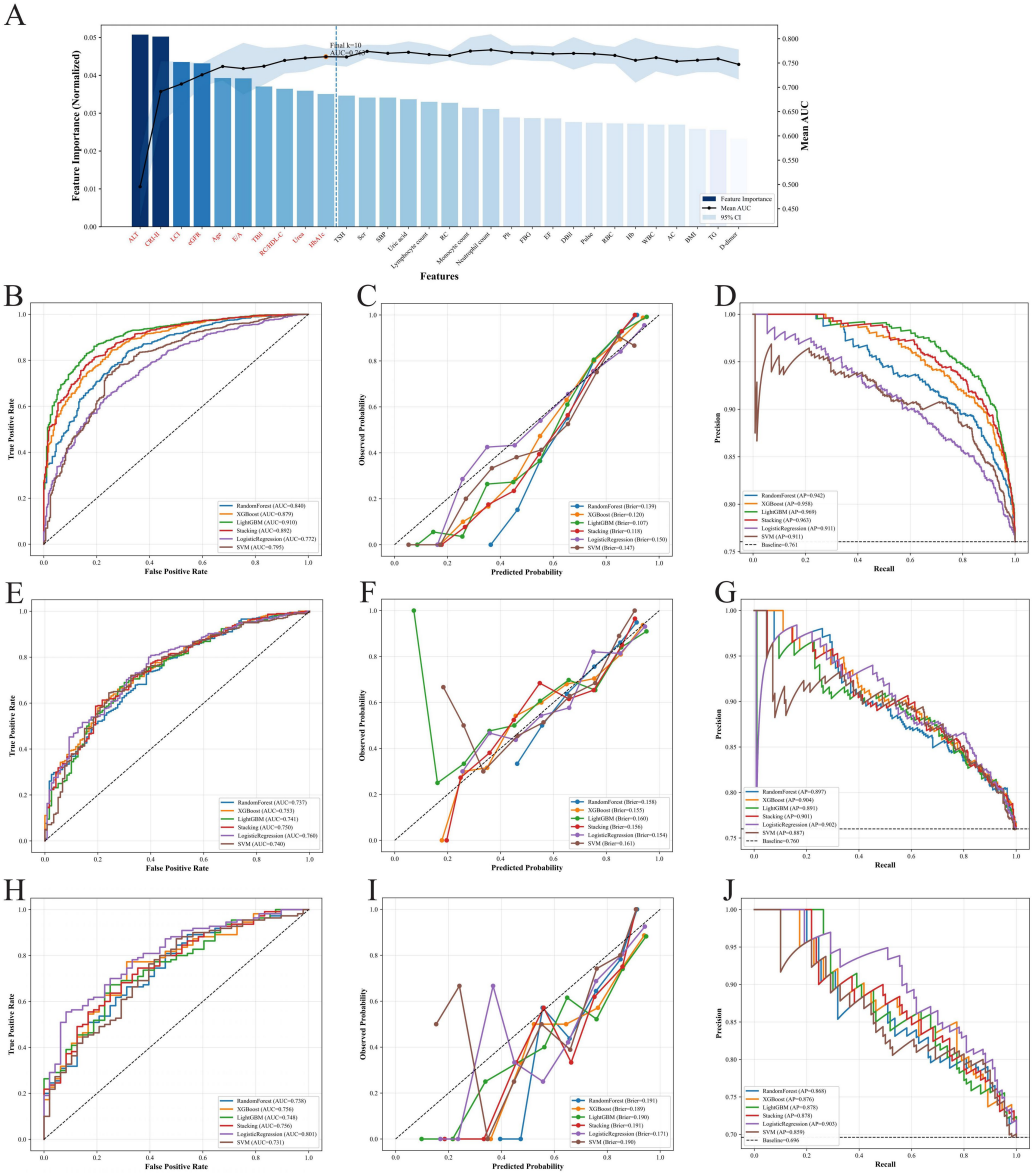
Feature selection process and comprehensive performance evaluation of machine learning models. **(A)** The sequential forward selection (SFS) process identifying the top 10 optimal features (highlighted in dark blue). **(B–J)** Performance comparison of six models across the training (top row), internal test (middle row), and external validation (bottom row) sets. The columns display ROC curves (left), calibration curves (center), and precision-recall curves (right). AUC, area under the curve; AP, average precision.

To reduce redundancy among the initially screened variables, we performed correlation-based clustering, with results shown in Supplementary Figure S3. Subsequent SFS indicated a marked improvement in discrimination as additional variables were incorporated. Subsequently, sequential forward selection (SFS) was applied to rank candidate variables according to their contribution to model performance, and the top 10 ranked variables were ultimately selected. The mean AUC achieved by five-fold cross-validation using these 10 variables was 0.763 (Supplementary Table S3). The final top 10 features were alanine aminotransferase (ALT), CRI-II, LCI, estimated glomerular filtration rate (eGFR), age, early-to-late diastolic transmitral flow velocity ratio (E/A), total bilirubin (TBil), RC/HDL-C, urea, and glycated hemoglobin (HbA1c) (Figure 3A). Consistently, intrinsic feature importance analyses of the three base learners underpinning the Stacking model (random forest, XGBoost, and LightGBM) showed broadly similar patterns, with substantial overlap in the top-ranked predictors across algorithms, further supporting the robustness of the selected feature set (Supplementary Figure S4).

Using these 10 variables, we systematically compared six machine learning models (Table 3; Figures 3B–J). Given that the Stacking model integrated multiple tree-based learners and showed competitive and relatively balanced performance across datasets, it was selected as the final classification model because of its greater robustness and stability. DCA further demonstrated that the Stacking model provided favorable net clinical benefit across a broad range of threshold probabilities (Supplementary Figure S5). In addition, confusion matrices across datasets (Supplementary Figure S6) and five-fold cross-validated ROC curves in the training set (Supplementary Figure S7) showed consistent discriminatory performance, further supporting the robustness of the final model.

**Table 3:** Performance of machine learning models in the training, internal test, and external validation sets.

Data set	Model	Threshold	AUC	AP	Accuracy	Sensitivity	Specificity	PPV	NPV	F1 Score	Brier score
Train	RF	0.690	0.840	0.942	0.804	0.856	0.642	0.883	0.583	0.869	0.139
	XGBoost	0.680	0.879	0.958	0.821	0.853	0.720	0.906	0.607	0.879	0.120
	LightGBM	0.690	0.910	0.969	0.848	0.863	0.799	0.932	0.648	0.896	0.107
	Stacking	0.720	0.892	0.963	0.827	0.853	0.746	0.914	0.615	0.883	0.118
	LR	0.640	0.772	0.911	0.767	0.851	0.498	0.843	0.513	0.847	0.150
	SVM	0.710	0.795	0.911	0.788	0.867	0.538	0.856	0.560	0.861	0.147
Test	RF	0.690	0.737	0.897	0.746	0.834	0.467	0.832	0.471	0.833	0.158
	XGBoost	0.680	0.753	0.904	0.740	0.824	0.475	0.832	0.460	0.828	0.155
	LightGBM	0.690	0.741	0.891	0.736	0.824	0.458	0.828	0.451	0.826	0.160
	Stacking	0.720	0.750	0.901	0.740	0.821	0.483	0.834	0.460	0.828	0.156
	LR	0.640	0.760	0.902	0.756	0.855	0.442	0.829	0.491	0.842	0.154
	SVM	0.710	0.740	0.887	0.742	0.829	0.467	0.831	0.463	0.830	0.161
Valid	RF	0.690	0.738	0.868	0.747	0.918	0.354	0.765	0.654	0.835	0.191
	XGBoost	0.680	0.756	0.876	0.728	0.891	0.354	0.760	0.586	0.820	0.189
	LightGBM	0.690	0.748	0.878	0.728	0.891	0.354	0.760	0.586	0.820	0.190
	Stacking	0.720	0.756	0.878	0.747	0.909	0.375	0.769	0.643	0.833	0.191
	LR	0.640	0.801	0.903	0.759	0.918	0.396	0.777	0.679	0.842	0.171
	SVM	0.710	0.731	0.859	0.753	0.864	0.500	0.798	0.615	0.830	0.190

AUC, area under the receiver operating characteristic curve; AP, average precision; RF, random forest; XGBoost, extreme gradient boosting; LightGBM, light gradient boosting machine; LR, logistic regression; SVM, support vector machine; NPV, negative predictive value; PPV, positive predictive value.

### 3.4 Model interpretability analyses

**Figure 4:**
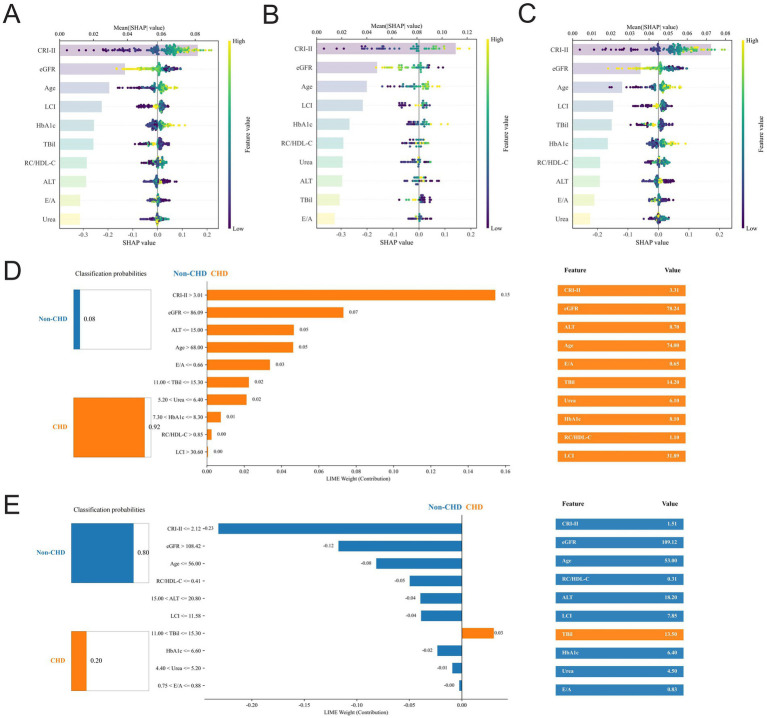
SHAP and LIME-based interpretability visualizations for the Stacking model. **(A–C)** SHAP summary plots depicting the distribution of SHA*p* values and global feature importance across the entire cohort **(A)**, the non-CHD group **(B)**, and the CHD group **(C)** within the internal test set. **(D, E)** LIME plots illustrating the specific feature contributions to the estimated probability of classification for a representative high-risk patient **(D)** and a low-risk patient **(E)** sampled from the internal test set. SHAP, Shapley Additive Explanations; LIME, Local Interpretable Model-agnostic Explanations; CHD, coronary heart disease.

We used complementary SHAP and LIME approaches to provide global and local, multidimensional explanations for the Stacking model (Figure 4). The SHAP beeswarm plot identified CRI-II, eGFR, age, and LCI as the four most influential features, followed by HbA1c, TBil, RC/HDL-C, ALT, E/A, and urea (Figure 4A). Higher values of CRI-II, age, LCI, and HbA1c were generally associated with positive SHAP values, indicating an increased contribution to CHD classification, whereas higher eGFR values were predominantly associated with negative SHAP values, suggesting a protective association. TBil, RC/HDL-C, ALT, E/A, and urea showed relatively smaller but still detectable contributions to model output. Stratified SHAP analyses showed consistent results in the non-CHD (Figure 4B) and CHD (Figure 4C) subgroups, with CRI-II, eGFR, age, and LCI consistently ranking as the top four features.

LIME-based local explanations further illustrated the individualized decision-making process of the model. For a representative CHD case with an estimated CHD probability of 0.92 (Figure 4D), the prediction was mainly driven by higher CRI-II, lower eGFR, and older age. In contrast, for a representative non-CHD case with an estimated non-CHD probability of 0.80 (Figure 4E), lower CRI-II, higher eGFR, younger age, and lower RC/HDL-C were the major contributors supporting the non-CHD classification. Overall, the main drivers identified by LIME were broadly consistent with the top features highlighted by SHAP, further supporting the robustness of the model interpretation.

**Figure 5:**
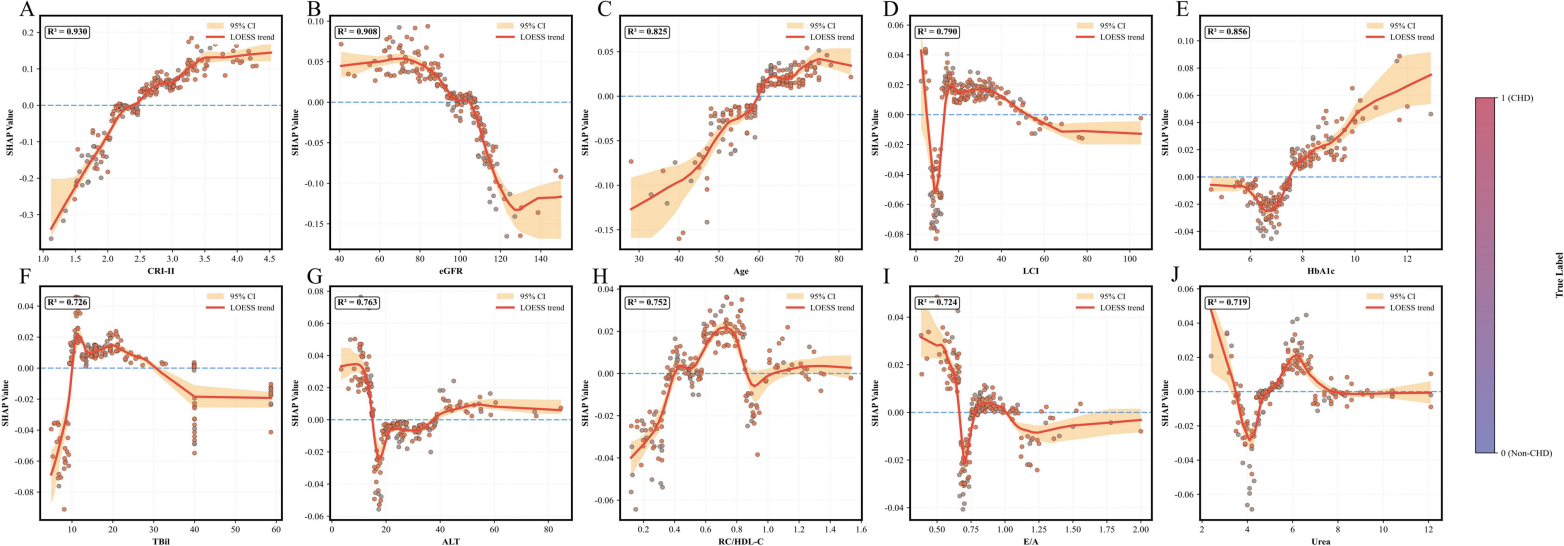
SHAP dependence plots illustrating the non-linear impacts of the features on coronary heart disease risk. The scatter plots display the specific SHAp value (y-axis) for each feature value (x-axis), with points colored by the true disease label (purple: non-CHD; red: CHD). The solid red line represents the smoothed trend fitted using LOESS regression, and the orange shaded area indicates the 95% confidence interval. The R^2^ value denotes the goodness of fit for the trend line. A SHAp value > 0 indicates a greater contribution toward CHD classification. **(A)** CRI-II, **(B)** eGFR, **(C)** Age, **(D)** LCI, **(E)** HbA1c, **(F)** TBil, **(G)** ALT, **(H)** RC/HDL-C, **(I)** E/A, and **(J)** Urea. SHAP, Shapley Additive Explanations; LOESS, locally estimated scatterplot smoothing; CI, confidence interval; CHD, coronary heart disease; CRI, Castelli risk index; eGFR, estimated glomerular filtration rate; LCI, lipoprotein combined index; HbA1c, glycated hemoglobin; TBil, total bilirubin; ALT, alanine aminotransferase; RC/HDL-C, remnant cholesterol to high-density lipoprotein cholesterol ratio; E/A, early-to-late diastolic transmitral flow velocity ratio.

In addition, LOESS-smoothed SHAP dependence plots (Figure 5) showed good goodness of fit (R^2^ = 0.719–0.930) and identified nonlinear relationships between the selected features and the model-assigned probability of CHD. CRI-II showed a progressive increase in SHAP values followed by a plateau, whereas eGFR showed an overall inverse association. Age was positively associated with SHAP values, and HbA1c showed an overall increasing trend at higher values. LCI, TBil, ALT, RC/HDL-C, E/A, and urea exhibited more complex fluctuating patterns, indicating potential threshold and saturation effects.

## 4 Discussion

This multicenter study, utilizing EMR data from two tertiary medical centers, represents the first systematic evaluation of the pivotal role of traditional and non-traditional lipid indices in identifying CHD among patients with T2DM and MASLD. We confirmed the superior discriminative utility of non-traditional lipid markers, particularly the CRI-II, within this high-risk population and successfully developed and validated a diagnostic classification model based on Stacking ensemble learning. These findings provide novel clinical evidence for understanding the role of the glucose-lipid-liver axis in promoting the progression of atherosclerosis.

In the pathological context of MASLD and T2DM coexistence, lipid metabolism disorders exhibit a high degree of complexity (23). A core finding of this study is that eight non-traditional lipid-derived indices (AIP, Non-HDL-C, AC, LCI, RC, RC/HDL-C, CRI-I, and CRI-II) were all significantly and positively correlated with CHD risk. Among these, CRI-II demonstrated the strongest independent association with CHD in the multivariable logistic regression model (OR = 2.394) and consistently ranked at the top of feature importance across multiple algorithms during the selection process. From a pathophysiological perspective, the bidirectional synergy between MASLD and T2DM exacerbates systemic insulin resistance, triggering a cascade of hepatic very low-density lipoprotein (VLDL) overproduction and the subsequent remodeling of LDL into highly atherogenic small dense particles (sdLDL) (24, 25). Previous research has confirmed that CRI-II (the LDL-C/HDL-C ratio) more comprehensively reflects the dynamic balance between atherogenic lipid particles and protective components (26). Our study further corroborated through Spearman correlation analysis that CRI-II showed the strongest positive correlation with the severity of coronary lesions (Gensini score; *ρ* = 0.302), whereas traditional lipid indicators such as TC, TG, and LDL-C showed no such statistical association, further underscoring the superiority of CRI-II in quantifying the burden of coronary artery disease.

Although evidence suggests that MASLD and T2DM act synergistically to drive the occurrence of CHD, their interactive impact within the context of complex lipid metabolism disorders warrants further exploration (27). While previous studies independently evaluated the clinical value of lipid indices in these conditions, such as identifying Non-HDL-C as closely related to long-term cardiovascular incidence in large cohorts or exploring the role of AIP in predicting coronary lesion severity, these studies often focused on single lipid entities or the general metabolic population (10, 28). Notably, through RCS analysis, this study revealed that in the specific high-risk cohort of MASLD combined with T2DM, indicators such as TG, RC, and AIP exhibited a non-linear “inverted U-shaped” association with CHD risk. This finding not only enriches the linear or J-shaped risk patterns observed in previous studies but also suggests the complexity of lipid pathogenic mechanisms within this group (28–30). This intricate relationship may reflect the pathogenic accumulation effect of residual cholesterol within specific thresholds, or it may indicate the presence of as-yet-unelucidated compensatory mechanisms or the potential impact of early clinical interventions at extremely high lipid levels.

The final classification model incorporated 10 key variables covering three clinically relevant domains, namely demographic characteristics, lipid-related indicators, and metabolic parameters. Its robustness and generalizability were supported by stratified five-fold cross-validation during model development, as well as consistent performance in both the internal test set and the external validation cohort. These variables are derived from routine clinical examinations, offering good accessibility, interpretability, and potential for widespread application. Regarding lipid metabolism, beyond the core factor CRI-II, the model introduces composite indices such as LCI and RC/HDL-C to characterize the non-traditional lipid burden beyond standard parameters. Previous research has shown that LCI is associated with the occurrence and severity of coronary artery disease and acute coronary syndromes (10, 31). RC/HDL-C, by characterizing residual cholesterol effects alongside HDL levels, more sensitively reflects residual lipid risk and correlates with coronary disease severity (32). The inclusion of these indicators suggests that residual risk may continue to drive coronary lesion progression even when baseline lipid control appears adequate (33, 34). Furthermore, the model integrates multidimensional metabolic and physiological parameters to augment its predictive performance. As a pivotal demographic variable, age represents a fundamental determinant of the initiation and progression of coronary atherosclerosis (35). In terms of glycemic metabolism, HbA1c reflects chronic glycemic variability, with elevated levels being strongly associated with coronary lesion complexity (36). Regarding the hepatorenal metabolic profile, eGFR and urea are pivotal markers of renal function that are closely associated with the risk and progression of CHD (37, 38), with eGFR serving as an independent predictor of coronary artery disease severity (39). Concurrently, the hepatic marker TBil functions as an endogenous antioxidant whose decline is independently associated with greater coronary artery disease severity (40). Meanwhile, ALT is frequently utilized as a clinical indicator of hepatic steatosis and insulin resistance, both of which are established independent contributors to the progression of cardiovascular disease (41). Finally, the inclusion of the E/A ratio adds hemodynamic and cardiac diastolic dimensions to the model, with abnormalities typically serving as early markers of myocardial impairment (42). Collectively, integrating these clinically accessible variables with traditional lipid indices supplements information on demographic characteristics, lipid metabolism, glycemic control, and hepatorenal metabolic status, thereby enhancing CHD identification and supporting more precise classification of patients with a high likelihood of prevalent CHD.

In the systematic evaluation of six machine learning algorithms, the Stacking ensemble model was established as the final classification model due to its superior performance in discrimination, calibration, and clinical utility. The model showed consistent performance in the internal test set and the external validation cohort, supporting its generalizability. A significant technical advantage of this study is the introduction of an explainable artificial intelligence (XAI) framework, combining global feature attribution via SHAP with local-level explanations via LIME (43). This approach maintains classification performance while enhancing the transparency of the decision-making process, providing evidence for the contribution direction and clinical interpretability of key variables. Specifically, SHAP quantifies the marginal contribution of features based on Shapley values to form a mathematically consistent global importance assessment; meanwhile, LIME provides local neighborhood explanations for individual samples, helping to identify individual risk drivers and thereby enhancing clinical understanding and trust.

While this study possesses significant advantages such as a multicenter design, systematic comparison of machine learning algorithms, independent external validation, and the implementation of model interpretability, several limitations warrant attention. First, because this was a retrospective cross-sectional study in which clinical variables and CHD status were collected simultaneously, the findings should be interpreted as associations rather than causal relationships, and no longitudinal or temporal inferences can be made. In addition, the influence of unmeasured variables, such as genetics and lifestyle factors, cannot be entirely excluded. Second, the diagnosis of MASLD relied on ultrasonography rather than the gold standard of liver biopsy. It is important to acknowledge that ultrasound has inherent limitations in the precise quantification and severity grading of hepatic steatosis, which may introduce potential bias in assessing the exact degree of the condition compared to histological examination. Third, the exclusion of patients using statins or triglyceride-lowering medications potentially biased the study population toward untreated or early-stage individuals, necessitating future research to validate the robustness of the model in populations receiving medical intervention. Fourth, although an external validation set was employed, it was limited to a relatively small Chinese cohort from a single center within the same national health system, which may restrict the global generalizability of the model. Future validation in larger and more diverse populations across different healthcare systems, geographical regions, and ethnic groups is needed to confirm the robustness of the model. Finally, because longitudinal follow-up data were unavailable, the present study could not assess the prognostic utility of the model for future cardiovascular outcomes.

## 5 Conclusion

In summary, our findings reveal a significant association between non-traditional lipid indices and the risk of CHD in patients with comorbid MASLD and T2DM. Among these indices, CRI-II emerged as the most effective marker for identifying CHD in this population, exhibiting a strong positive correlation with coronary lesion severity as quantified by the Gensini score. Furthermore, the Stacking ensemble model developed and validated in this study demonstrated robust discriminatory performance across both internal and external cohorts. This model serves as an efficient and practical clinical decision-support tool for individualized CHD identification in this high-risk metabolic population.

## Data Availability

The original contributions presented in the study are included in the article/Supplementary material, further inquiries can be directed to the corresponding authors.
