# Supplementary material for: Interpretable machine learning of non-traditional lipid indices for diagnostic classification of CHD in patients with comorbid MASLD and T2DM: a multicenter study
Source: Front Nutr. 2026 Apr 22;13:1803996. doi: 10.3389/fnut.2026.1803996 (PMC13144128; doi:10.3389/fnut.2026.1803996)
Supplement: Supplementary file 1 [file Data_Sheet_1.PDF]

Supplementary Materials

Supplementary Table S1. Baseline characteristics of the study population across training, internal test, and external validation cohorts. ***Pages 2-3***

Supplemental Table S2. Description of data types and missing values for each feature in Cohort I and Cohort II. ***Pages 4-5***

Supplemental Table S3. Sequential forward selection process for optimal feature subset identification based on LightGBM. ***Pages 6-7***

Supplemental Figure S1. Evaluation of covariate balance before and after propensity score matching. ***Page 8***

Supplemental Figure S2. Spearman correlation analysis between lipid indices and the severity of coronary artery stenosis quantified by the Gensini score. ***Page 9***

Supplemental Figure S3. Collinearity analysis and hierarchical clustering of the top 30 candidate features. ***Page 10***

Supplemental Figure S4. Comparison of feature importance rankings among the three tree-based machine learning models. ***Page 11***

Supplemental Figure S5. Decision curve analysis of the six machine learning models for predicting coronary heart disease. ***Page 12***

Supplemental Figure S6. Confusion matrices of the six machine learning models across the training, internal test, and external validation cohorts. ***Page 13***

Supplemental Figure S7. Receiver operating characteristic (ROC) curves for 5-fold cross-validation of the six machine learning models in the training set. ***Pages 14***

Supplementary Table S1. Baseline characteristics of the study population across training, internal test, and external validation cohorts.

Variables	Train (n=1165)	Test (n=500)	Valid (n=158)	P
CHD, n (%)				0.201
No	279 (23.9)	120 (24.0)	48 (30.4)	
Yes	886 (76.1)	380 (76.0)	110 (69.6)	
Hypertension, n (%)				0.485
No	351 (30.1)	158 (31.6)	42 (26.6)	
Yes	814 (69.9)	342 (68.4)	116 (73.4)	
Sex, n (%)				0.186
Female	731 (62.7)	301 (60.2)	88 (55.7)	
Male	434 (37.3)	199 (39.8)	70 (44.3)	
Smoking, n (%)				0.764
No	850 (83.9)	365 (73.0)	111 (70.3)	
Yes	315 (16.1)	135 (27.0)	47 (29.7)	
Age,years(mean (SD))	61.35 (9.17)	61.11 (8.80)	60.93 (10.98)	0.800
BMI,kg/m^2(mean (SD))	26.83 (3.20)	26.93 (3.26)	27.12 (3.13)	0.545
Pulse,bpm(mean (SD))	73.24 (11.51)	72.70 (10.57)	73.06 (12.29)	0.666
SBP,mmhg(mean (SD))	134.17 (16.56)	134.29 (16.59)	135.73 (16.24)	0.537
DBP,mmhg(mean (SD))	80.28 (9.85)	80.51 (9.96)	80.84 (9.23)	0.765
Monocyte count,10^9/L(mean (SD))	0.46 (0.14)	0.46 (0.15)	0.46 (0.15)	0.474
Neutrophil count,10^9/L(mean (SD))	3.80 (1.30)	3.82 (1.27)	4.09 (1.52)	0.031
Lymphocyte count,10^9/L(mean (SD))	2.01 (0.61)	2.01 (0.59)	1.96 (0.60)	0.565
WBC,10^9/L(mean (SD))	6.44 (1.61)	6.47 (1.62)	6.68 (1.82)	0.245
RBC,10^{12}/L(mean (SD))	4.59 (0.49)	4.60 (0.50)	4.54 (0.51)	0.458
Plt,10^9/L(mean (SD))	220.10 (55.41)	219.40 (53.51)	201.53 (56.17)	<0.001
Hb,g/L(mean (SD))	138.56 (15.06)	138.34 (14.66)	135.72 (15.27)	0.081
FBG,mmol/L(mean (SD))	7.01 (2.29)	6.84 (2.14)	7.37 (2.44)	0.038
HbA1c,%(mean (SD))	7.63 (1.36)	7.61 (1.36)	7.66 (1.46)	0.900
LDL-C,mmol/L(mean (SD))	2.56 (0.68)	2.54 (0.68)	2.66 (0.71)	0.146
TC,mmol/L(mean (SD))	4.19 (0.92)	4.15 (0.91)	4.37 (1.05)	0.029
TG,mmol/L(mean (SD))	1.96 (0.91)	2.00 (0.91)	2.19 (1.41)	0.025
HDL-C,mmol/L(mean (SD))	1.01 (0.21)	0.98 (0.20)	1.00 (0.22)	0.058
ALT,U/L(mean (SD))	24.46 (13.87)	25.07 (14.90)	28.51 (18.51)	0.005
TBil, μ mol/L(mean (SD))	19.60 (13.53)	19.46 (12.40)	16.69 (6.75)	0.026
DBil, μ mol/L(mean (SD))	3.86 (2.32)	3.90 (2.33)	3.03 (1.22)	<0.001
ALB,g/L(mean (SD))	41.66 (3.29)	41.70 (3.50)	41.14 (3.25)	0.163
Urea,mmol/L(mean (SD))	5.53 (1.66)	5.54 (1.63)	5.28 (1.62)	0.178
Uric acid, μ mol/L(mean (SD))	320.62 (85.53)	317.36 (88.51)	326.21 (82.63)	0.512
Scr, μ mol/L(mean (SD))	62.06 (13.82)	62.74 (14.24)	60.16 (13.75)	0.128
eGFR,mL/min/1.73 m^2 (mean (SD))	96.39 (17.43)	95.44 (18.88)	98.23 (11.66)	0.204
NT-proBNP,pg/mL(median (IQR))	55.00 [28.00,	51.50 [27.00,	113.50 [53.75,	<0.001

	110.00]	114.00]	914.00]	
hs-cTnI,pg/L(median (IQR))	3.26 [2.26, 5.34]	3.56 [2.18, 5.50]	3.64 [1.19, 8.17]	0.589
D-dimer,mg/L(median (IQR))	230.00 [160.00, 350.00]	220.00 [160.00, 320.00]	261.75 [176.28, 392.95]	0.035
PT,s(mean (SD))	13.76 (1.78)	13.78 (1.90)	14.63 (1.06)	<0.001
PT ratio (mean (SD))	1.02 (0.08)	1.02 (0.09)	1.06 (0.11)	<0.001
INR (mean (SD))	1.02 (0.09)	1.02 (0.10)	1.06 (0.13)	<0.001
APTT,s(mean (SD))	30.80 (3.32)	30.74 (3.25)	31.33 (4.79)	0.152
TSH,mIU/L(mean (SD))	3.02 (2.39)	2.98 (2.33)	3.61 (6.23)	0.045
LA,mm(mean (SD))	36.00 (3.54)	35.83 (3.68)	35.64 (3.62)	0.397
IVSD,mm(mean (SD))	9.59 (1.94)	9.53 (1.86)	9.99 (1.96)	0.026
LVIDd,mm(mean (SD))	45.59 (3.38)	45.63 (3.44)	46.39 (3.40)	0.020
LVPWd,mm(mean (SD))	8.76 (1.08)	8.80 (1.04)	9.04 (1.23)	0.008
E/A (mean (SD))	0.82 (0.27)	0.83 (0.28)	0.86 (0.28)	0.211
EF,%(mean (SD))	65.38 (6.59)	65.90 (6.43)	63.46 (8.30)	<0.001
AIP (mean (SD))	0.26 (0.22)	0.28 (0.21)	0.29 (0.25)	0.061
Non-HDL-C,mmol/L(mean (SD))	3.18 (0.83)	3.16 (0.80)	3.37 (0.97)	0.019
AC (mean (SD))	3.22 (0.85)	3.28 (0.82)	3.47 (1.10)	0.004
CRI-I (mean (SD))	4.22 (0.85)	4.28 (0.82)	4.47 (1.10)	0.004
CRI-II (mean (SD))	2.58 (0.67)	2.62 (0.65)	2.73 (0.77)	0.026
LCI (mean (SD))	22.90 (15.66)	23.17 (15.37)	30.87 (38.80)	<0.001
RC,mmol/L(mean (SD))	0.63 (0.30)	0.62 (0.31)	0.71 (0.33)	0.004
RC/HDL-C (mean (SD))	0.65 (0.33)	0.66 (0.34)	0.74 (0.39)	0.004

Data are presented as mean (SD) for normally distributed continuous variables, median [IQR] for non-normally distributed continuous variables, or n (%) for categorical variables. P-values were calculated using one-way ANOVA or Kruskal-Wallis H test for continuous variables, and Chi-square test for categorical variables to compare differences among the three groups. Abbreviations: CHD, coronary heart disease; BMI, body mass index; SBP, systolic blood pressure; DBP, diastolic blood pressure; WBC, white blood cell; RBC, red blood cell; Plt, platelets; Hb, hemoglobin; FBG, fasting blood glucose; HbA1c, glycated hemoglobin; LDL-C, low-density lipoprotein cholesterol; TC, total cholesterol; TG, triglycerides; HDL-C, high-density lipoprotein cholesterol; ALT, alanine aminotransferase; TBil, total bilirubin; DBil, direct bilirubin; ALB, albumin; Scr, serum creatinine; eGFR, estimated glomerular filtration rate; NT-proBNP, N-terminal pro-B-type natriuretic peptide; hs-cTnI, high-sensitivity cardiac troponin I; PT, prothrombin time; INR, international normalized ratio; APTT, activated partial thromboplastin time; TSH, thyroid stimulating hormone; LA, left atrial diameter; IVSD, interventricular septal thickness at diastole; LVIDd, left ventricular internal diameter at diastole; LVPWd, left ventricular posterior wall thickness at diastole; EF, ejection fraction; AIP, atherogenic index of plasma; Non-HDL-C, non-high-density lipoprotein cholesterol; AC, atherogenic coefficient; CRI, cardiac risk index; LCI, lipid comprehensive index; RC, remnant cholesterol.

Supplemental Table S2. Description of data types and missing values for each feature in Cohort I and Cohort II.

Features	Data Types	Missing Values	
		Cohort I(n=1665)	Cohort II (n=158)
**Demographics**			
Age	Continuous	0 (0)	0 (0)
Sex	Categorical	0 (0)	0 (0)
Body mass index	Continuous	0 (0)	3 (1.90%)
Smoking	Categorical	0 (0)	0 (0)
Hypertension	Categorical	0 (0)	0 (0)
CHD	Categorical	0 (0)	0 (0)
**Vital signs**			
Pulse	Continuous	2 (0.12%)	0 (0)
SBP	Continuous	26 (1.56%)	0 (0)
DBP	Continuous	15 (0.90%)	0 (0)
**Hematology**			
White blood cells	Continuous	6 (0.36%)	10 (6.33%)
Red blood cells	Continuous	6 (0.36%)	10 (6.33%)
Platelets	Continuous	6 (0.36%)	10 (6.33%)
Hemoglobin	Continuous	6 (0.36%)	10 (6.33%)
Monocyte count	Continuous	6 (0.36%)	10 (6.33%)
Neutrophil count	Continuous	6 (0.36%)	10 (6.33%)
Lymphocyte count	Continuous	6 (0.36%)	10 (6.33%)
**Glycemic indices**			
FBG	Continuous	34 (2.04%)	6 (3.80%)
HbA1c	Continuous	149 (8.95%)	18 (11.39%)
**Lipids**			
LDL-C	Continuous	0 (0)	0 (0)
TC	Continuous	0 (0)	0 (0)
TG	Continuous	0 (0)	0 (0)
HDL-C	Continuous	0 (0)	0 (0)
Non-HDL-C	Continuous	0 (0)	0 (0)
RC	Continuous	0 (0)	0 (0)
RC/HDL-C	Continuous	0 (0)	0 (0)
AIP	Continuous	0 (0)	0 (0)
AC	Continuous	0 (0)	0 (0)
CRI-I	Continuous	0 (0)	0 (0)
CRI-II	Continuous	0 (0)	0 (0)
LCI	Continuous	0 (0)	0 (0)
**Liver function**			
ALT	Continuous	8 (0.48%)	5 (3.16%)
TBil	Continuous	301 (18.08%)	16 (10.13%)
DBil	Continuous	362 (21.74%)	20 (12.66%)
ALB	Continuous	296 (17.78%)	18 (11.39%)
**Renal function**			

Urea	Continuous	80 (4.80%)	14 (8.86%)
Uric acid	Continuous	33 (1.98%)	15 (9.49%)
Scr	Continuous	11 (0.66%)	12 (7.59%)
eGFR	Continuous	11 (0.66%)	12 (7.59%)
**Cardiac biomarkers**			
NT-proBNP	Continuous	217 (13.03%)	16 (10.13%)
hs-cTnI	Continuous	84 (5.05%)	10 (6.96%)
**Coagulation**			
D-dimer	Continuous	32 (1.92%)	8 (5.06%)
PT(s)	Continuous	42 (2.52%)	15 (9.49%)
PT ratio	Continuous	42 (2.52%)	11 (6.96%)
INR	Continuous	42 (2.52%)	11 (6.96%)
APTT	Continuous	209 (12.55%)	39 (24.68%)
**Thyroid function**			
TSH	Continuous	168 (10.09%)	30 (18.99%)
**Echocardiography**			
LA	Continuous	253 (15.20%)	10 (6.33%)
IVSD	Continuous	253 (15.20%)	10 (6.33%)
LVIDd	Continuous	253 (15.20%)	10 (6.33%)
LVPWd	Continuous	253 (15.20%)	10 (6.33%)
EF	Continuous	253 (15.20%)	10 (6.33%)
E/A	Continuous	253 (15.20%)	14 (8.86%)

Data regarding missing values are presented as number (percentage). Abbreviations: CHD, coronary heart disease; SBP, systolic blood pressure; DBP, diastolic blood pressure; FBG, fasting blood glucose; HbA1c, glycated hemoglobin; LDL-C, low-density lipoprotein cholesterol; TC, total cholesterol; TG, triglycerides; HDL-C, high-density lipoprotein cholesterol; Non-HDL-C, non-high-density lipoprotein cholesterol; RC, remnant cholesterol; AIP, atherogenic index of plasma; AC, atherogenic coefficient; CRI, cardiac risk index; LCI, lipid comprehensive index; ALT, alanine aminotransferase; TBil, total bilirubin; DBil, direct bilirubin; ALB, albumin; Scr, serum creatinine; eGFR, estimated glomerular filtration rate; NT-proBNP, N-terminal pro-B-type natriuretic peptide; hs-cTnI, high-sensitivity cardiac troponin I; PT, prothrombin time; INR, international normalized ratio; APTT, activated partial thromboplastin time; TSH, thyroid stimulating hormone; LA, left atrial diameter; IVSD, interventricular septal thickness at diastole; LVIDd, left ventricular internal diameter at diastole; LVPWd, left ventricular posterior wall thickness at diastole; EF, ejection fraction; E/A, ratio of early to late diastolic transmitral flow velocity.

Supplemental Table S3. Sequential forward selection process for optimal feature subset identification based on LightGBM.

Step	Feature	Mean_ROC	Fold_1_ROC	Fold_2_ROC	Fold_3_ROC	Fold_4_ROC	Fold_5_ROC	Importance	95%CI
1	ALT	0.495316	0.438710654	0.484009282	0.457728006	0.556345843	0.539785495	5.075	0.495 (0.432–0.559)
2	CRI-II	0.691271	0.707526231	0.757314366	0.709846651	0.635088781	0.646578141	5.023	0.691 (0.629–0.754)
3	LCI	0.706829	0.742937853	0.715899919	0.709493543	0.644774011	0.721041879	4.351	0.707 (0.661–0.753)
4	eGFR	0.725758	0.732344633	0.744148507	0.737893462	0.696529459	0.717875383	4.317	0.726 (0.702–0.749)
5	Age	0.742893	0.746065375	0.743896287	0.776684826	0.711965295	0.735852911	3.929	0.743 (0.714–0.772)
6	E/A	0.738486	0.773809524	0.757717918	0.770833333	0.674132365	0.715934627	3.92	0.738 (0.685–0.792)
7	TBil	0.743407	0.773305085	0.769774011	0.751109766	0.707828894	0.715015322	3.705	0.743 (0.706–0.781)
8	RC/HDL-C	0.75533	0.756759483	0.776634383	0.775928168	0.728410008	0.738917263	3.645	0.755 (0.728–0.782)
9	Urea	0.760306	0.772497982	0.772296207	0.783292978	0.729418886	0.744024515	3.593	0.760 (0.732–0.788)
10	HbA1c	0.762978	0.750807103	0.76937046	0.781476998	0.749495561	0.763738509	3.507	0.763 (0.746–0.780)
11	TSH	0.762412	0.766242938	0.787933818	0.759281679	0.751412429	0.747191011	3.464	0.762 (0.743–0.782)
12	Scr	0.773899	0.771791768	0.783242534	0.759281679	0.768664245	0.786516854	3.412	0.774 (0.760–0.788)
13	SBP	0.769921	0.785108959	0.77905569	0.774213075	0.754741727	0.75648621	3.412	0.770 (0.753–0.787)
14	Uric acid	0.772086	0.781476998	0.79287732	0.774414851	0.744652946	0.76700715	3.369	0.772 (0.750–0.795)
15	Lymphocyte count	0.767506	0.765133172	0.795399516	0.765637611	0.75232042	0.759039837	3.3	0.768 (0.747–0.788)
16	RC	0.765324	0.761904762	0.778551251	0.761097659	0.759180791	0.765883555	3.274	0.765 (0.756–0.775)
17	Monocyte count	0.77453	0.798325262	0.792574657	0.772800646	0.740920097	0.768028601	3.145	0.775 (0.746–0.803)
18	Neutrophil count	0.776994	0.786117837	0.80306699	0.763619855	0.738498789	0.793667007	3.11	0.777 (0.745–0.809)
19	Plt	0.771653	0.770682002	0.785714286	0.764426957	0.757566586	0.779877426	2.886	0.772 (0.758–0.786)
20	FBG	0.770545	0.767251816	0.790052462	0.77491929	0.756355932	0.764147089	2.869	0.771 (0.755–0.786)
21	EF	0.768758	0.782485876	0.778853914	0.76937046	0.753631961	0.759448417	2.861	0.769 (0.753–0.784)
22	DBil	0.769794	0.786117837	0.804277643	0.772296207	0.740415658	0.745863126	2.766	0.770 (0.736–0.803)
23	Pulse	0.76879	0.774414851	0.780972559	0.780468119	0.743845843	0.764249234	2.749	0.769 (0.750–0.788)
24	RBC	0.765631	0.76523406	0.77905569	0.778652139	0.745560936	0.759652707	2.731	0.766 (0.748–0.783)
25	Hb	0.755541	0.76937046	0.798022599	0.768260694	0.699152542	0.742900919	2.723	0.756 (0.710–0.802)

26	WBC	0.761039	0.761501211	0.787530266	0.7781477	0.731335755	0.746680286	2.697	0.761 (0.733–0.789)
27	AC	0.753447	0.748385795	0.785714286	0.770782889	0.725988701	0.736363636	2.697	0.753 (0.723–0.784)
28	BMI	0.755712	0.757667474	0.78551251	0.750605327	0.730125101	0.7546476	2.585	0.756 (0.731–0.780)
29	TG	0.758606	0.744652946	0.791565779	0.757465698	0.765738499	0.73360572	2.559	0.759 (0.731–0.786)
30	D-dimer	0.747133	0.720338983	0.78551251	0.744047619	0.732243745	0.753524004	2.326	0.747 (0.716–0.778)

Features are listed in the order they were added to the model. The "Importance" column represents the normalized percentage contribution of each feature derived from the LightGBM model.

The Mean ROC and 95% CI were calculated based on 5-fold cross-validation.

Supplemental Figure S1. Evaluation of covariate balance before and after propensity score matching.

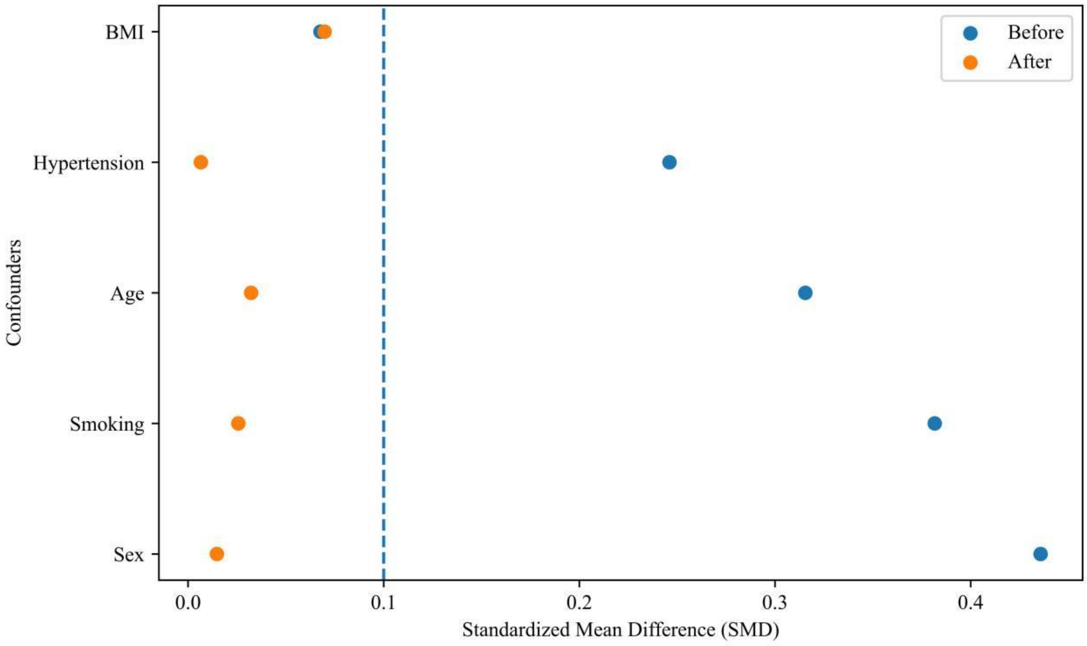

Supplemental Figure S2. Spearman correlation analysis between lipid indices and the severity of coronary artery stenosis quantified by the Gensini score.

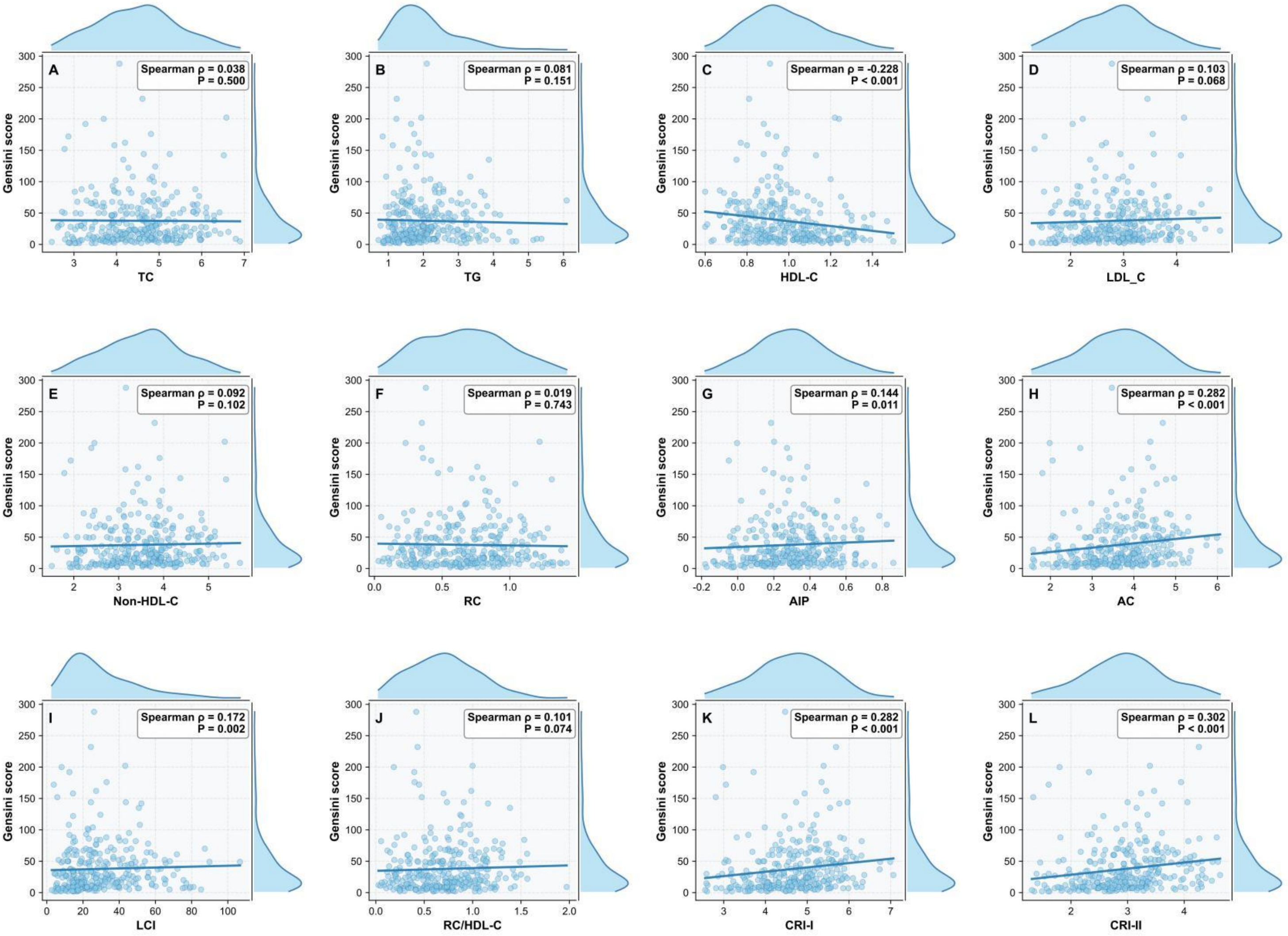

Supplemental Figure S3. Collinearity analysis and hierarchical clustering of the top 30 candidate features.

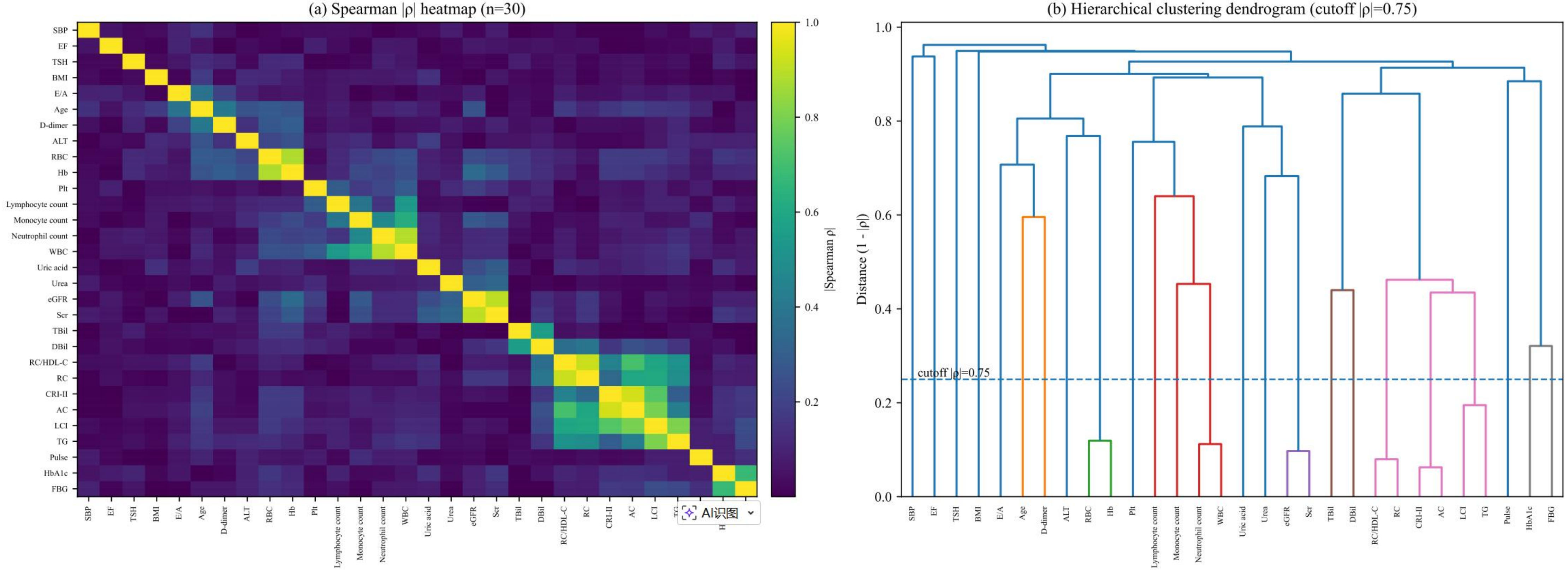

Supplemental Figure S4. Comparison of feature importance rankings among the three tree-based machine learning models.

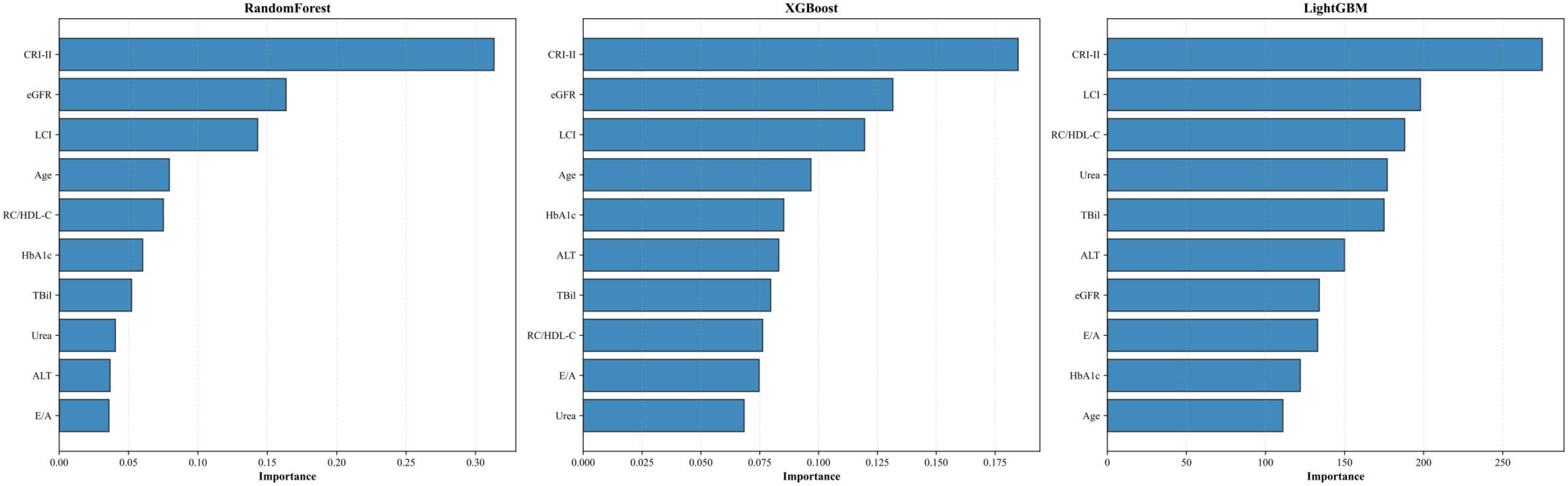

Supplemental Figure S5. Decision curve analysis of the six machine learning models for predicting coronary heart disease.

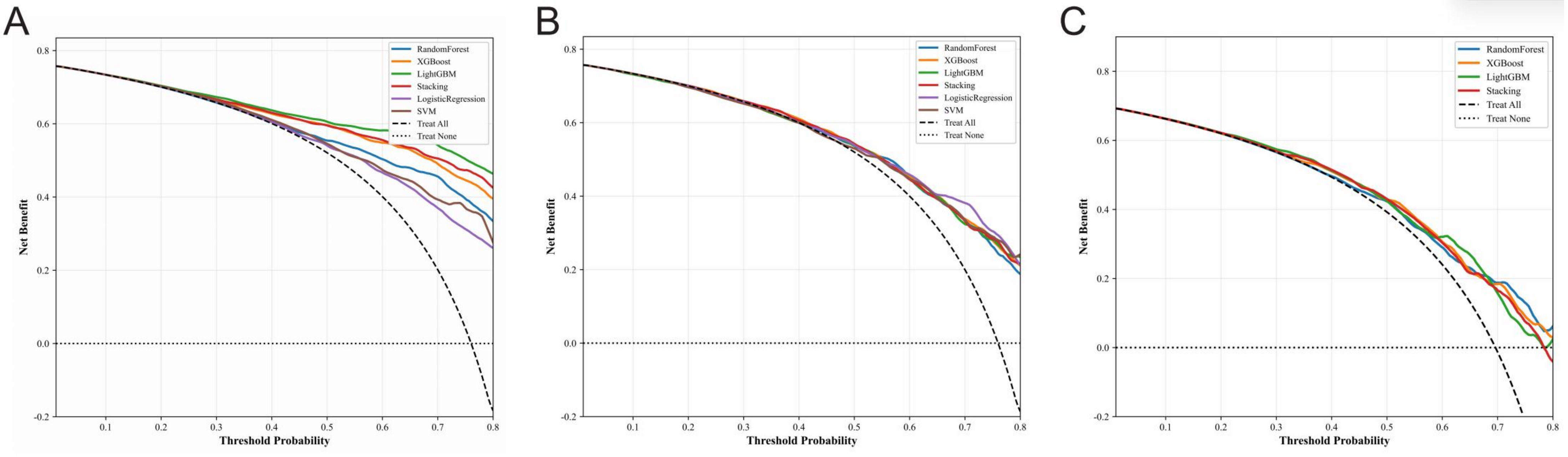

Supplemental Figure S6. Confusion matrices of the six machine learning models across the training, internal test, and external validation cohorts.

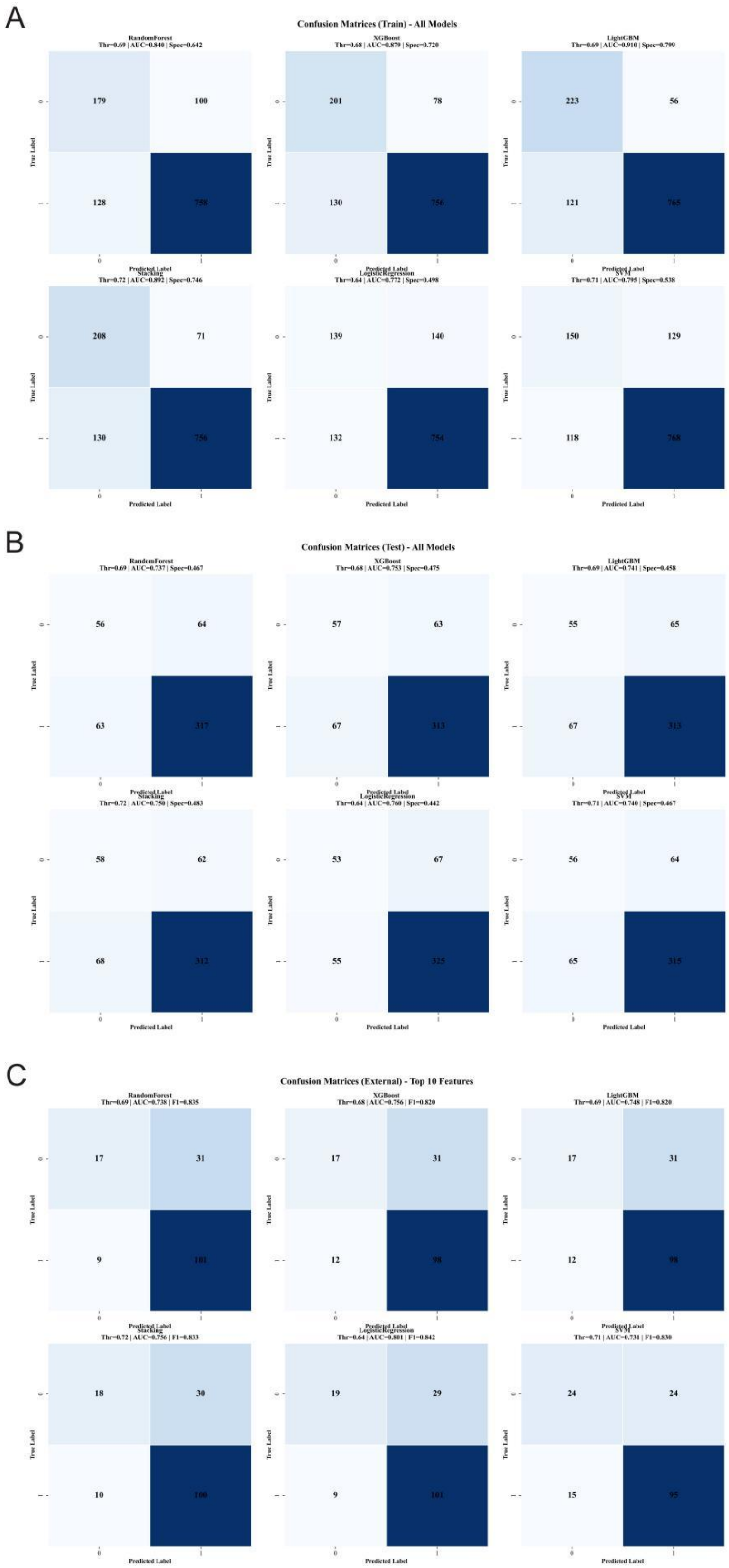

Supplemental Figure S7. Receiver operating characteristic (ROC) curves for 5-fold cross-validation of the six machine learning models in the training set.

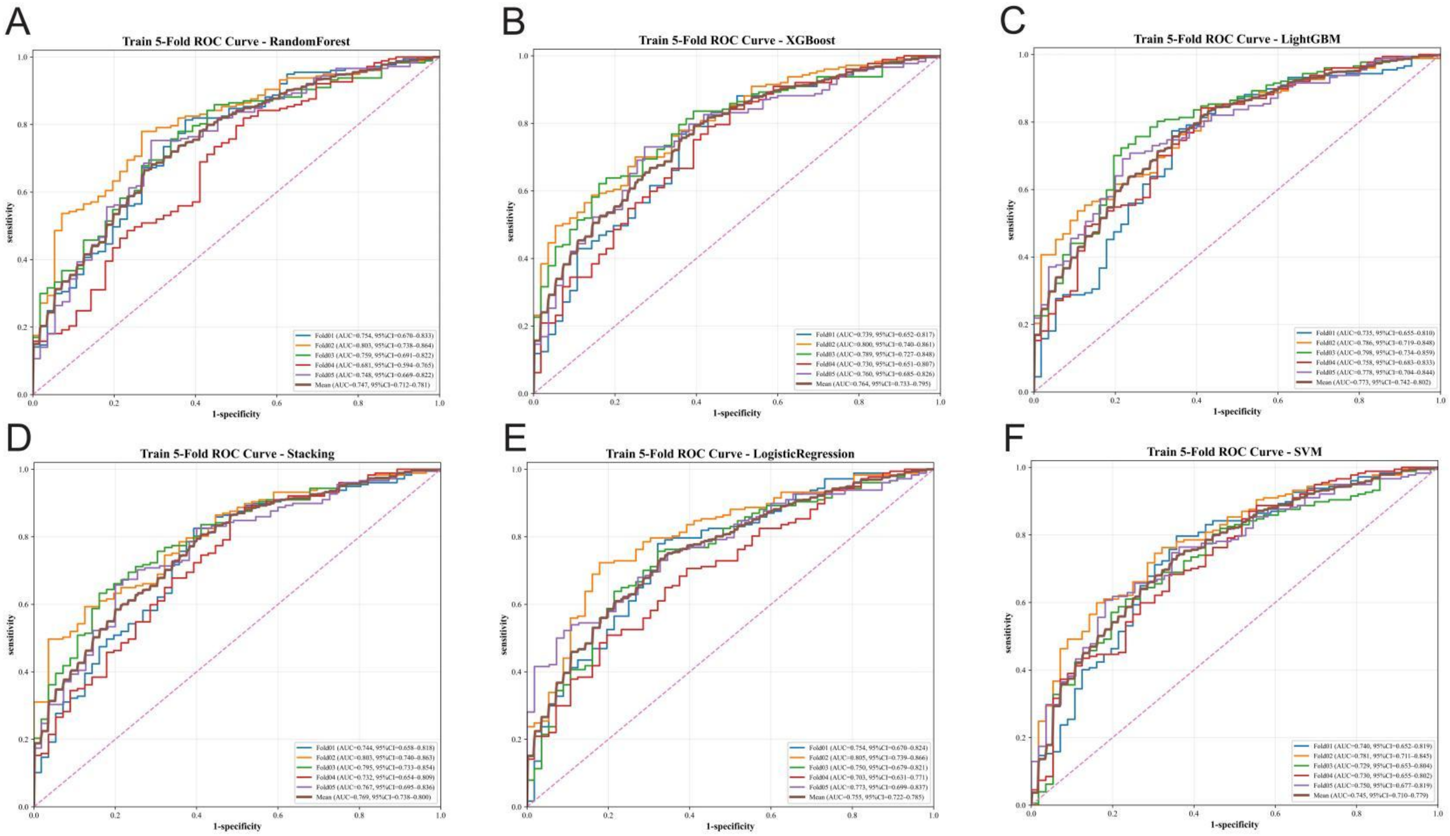

The figure displays the ROC curves for each of the 5 cross-validation folds (colored lines) and the mean ROC curve (bold brown line) for the following models: (A) Random Forest, (B) XGBoost, (C) LightGBM, (D) Stacking, (E) Logistic Regression, and (F) Support Vector Machine. The shaded gray area represents the standard deviation around the mean ROC curve. The mean area under the curve (AUC) and its 95% confidence interval are provided in the legend of each panel, demonstrating the stability of model performance. Abbreviations: ROC, receiver operating characteristic; AUC, area under the curve.
